# Autonomic nervous system dysfunction in critically ill cancer patients: Hemodynamic consequences and multimodal assessment strategies^☆^

**DOI:** 10.1016/j.cpt.2026.08.001

**Published:** 2026-10-21

**Authors:** Shiyi Gong, Yun Chu, Xiaoting Wang

**Affiliations:** Department of Critical Care Medicine, Peking Union Medical College Hospital, CAMS, Beijing, 100730, China

**Keywords:** Autonomic nervous system, Critical illness, Cancer, Host-organ unregulated response, Hemodynamic monitoring, Heart rate variability, Multimodal monitoring

## Abstract

In critically ill cancer patients, autonomic nervous system (ANS) dysfunction may contribute to hemodynamic instability and organ dysfunction and may be exacerbated by malignancy-related injury and anticancer treatment. This narrative review summarizes conventional methods for assessing ANS function, including heart rate variability (HRV), blood pressure variability (BPV), baroreflex sensitivity (BRS), and cardiovascular autonomic reflex testing, together with emerging approaches such as high-resolution multimodal monitoring (MMM) and artificial intelligence (AI). Host-organ unregulated response (HOUR) framework is used to integrate interactions among autonomic, neuroendocrine, inflammatory, immune, coagulation, metabolic, bioenergetic, and microcirculatory dysregulation. Collectively, these approaches may facilitate real-time assessment of autonomic function and tissue perfusion, thereby supporting risk stratification and individualized management in critically ill cancer patients.

## Highlights

• Autonomic nervous system (ANS) dysfunction may contribute to hemodynamic instability and organ dysfunction within the host-organ unregulated response (HOUR) framework
• ANS function can be evaluated using complementary cardiovascular autonomic assessment modalities.
• Complementary assessment methods include heart rate variability (HRV), blood pressure variability (BPV), baroreflex sensitivity (BRS) and standardized cardiovascular autonomic reflex testing
• High-resolution multimodal monitoring (MMM) and artificial intelligence (AI) may improve physiological assessment and risk stratification, although cancer-specific validation remains necessary. Management should prioritize the underlying cause, individualized supportive care, and disease-specific therapy

## Graphical abstract

**Figure:**
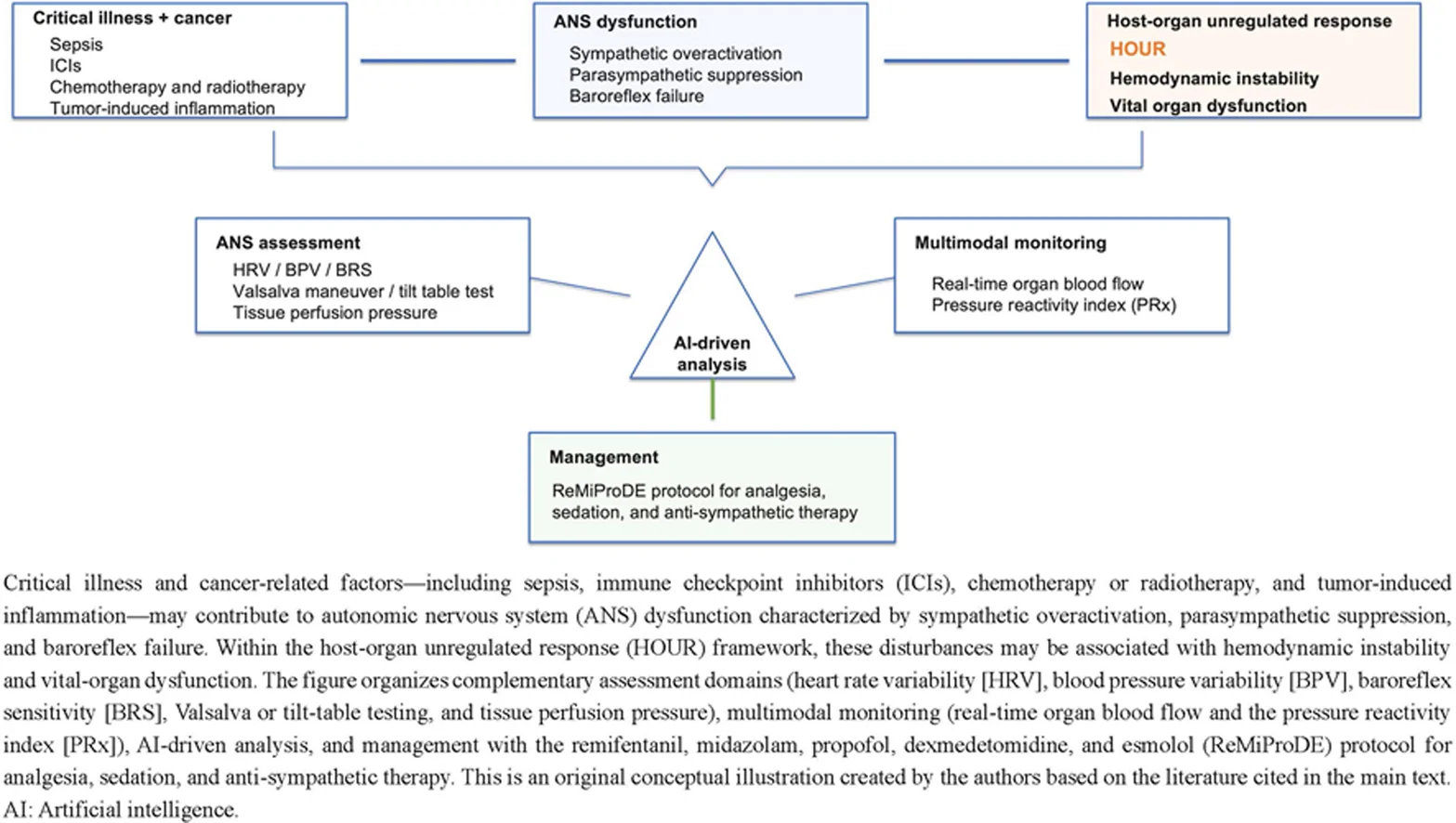

## Introduction

The circulatory and respiratory systems work together to deliver oxygen and remove carbon dioxide.^1^ Under physiological conditions, autonomic nervous system (ANS)-mediated regulation helps match oxygen delivery to tissue demand and maintain hemodynamic homeostasis.^2^ In critical illness, impaired cardiovascular and respiratory function can reduce oxygen delivery, contributing to tissue hypoperfusion, cellular injury, and multiple organ dysfunction syndrome (MODS).^3^

In critically ill cancer patients, this pathophysiological cascade may be more pronounced. Malignancy and treatments for cancer can directly damage the ANS and trigger systemic inflammation and metabolic imbalance, further impairing autonomic regulation.^4^^,^^5^ This malignancy-associated dysautonomia can aggravate hemodynamic instability, microcirculatory dysfunction, and progression to MODS.^6^

Point-of-care ultrasound (POCUS) provides serial bedside assessment of organ structure, function, and selected blood-flow variables.^7^^,^^8^ Because intermittent examinations may not capture transient waveform abnormalities, POCUS can be complemented by continuous synchronized physiological monitoring when clinically indicated.^9^ Artificial intelligence (AI)-based analysis may assist with complex time-series interpretation.^10^ This narrative review examines established and emerging approaches to ANS assessment in critically ill cancer patients.

### Literature search strategy

PubMed, Embase, and Web of Science were searched iteratively for English-language publications from January 2020 through May 2026. Seminal pre-2020 studies were also included when essential to define established physiological concepts or assessment methods. Search concepts included autonomic dysfunction or dysautonomia, cancer or neoplasms, critical illness or intensive care, hemodynamic monitoring, heart rate variability (HRV), blood pressure variability (BPV), baroreflex sensitivity (BRS), chemotherapy, radiotherapy, immunotherapy, and targeted therapy. Relevant publications were selected for their relevance to ANS function, organ-specific hemodynamic consequences, and clinical applicability to critically ill cancer patients. Where direct evidence in critically ill cancer patients was limited, selected evidence from related critical-care, neurocritical-care, perioperative oncology, cancer-survivorship, and physiological research was used to contextualize the discussion. Supplementary Table 1 presents representative clinical studies included in this narrative review.

## Physiological basis of the autonomic nervous system (ANS) and hemodynamic homeostasis

Hemodynamic homeostasis is the dynamic balance between oxygen delivery and tissue metabolic demand that preserves vital organ function. The ANS is the primary regulator of this balance through continuous cardiovascular adjustments.

The ANS maintains hemodynamic homeostasis through sympathetic–parasympathetic interactions. Sympathetic activation increases vascular tone through α_1_-adrenergic signaling and increases heart rate and myocardial contractility predominantly through β_1_-adrenergic signaling; parasympathetic vagal activity primarily slows sinus rate and atrioventricular conduction.^11^^,^^12^

Acute stress activates coordinated, but distinct, sympathoadrenal and hypothalamic-pituitary-adrenal (HPA) responses. Sympathetic nerves and the adrenal medulla release catecholamines, whereas HPA-axis activation increases glucocorticoid secretion.^13^^,^^14^ Catecholamines can temporarily support cardiac output and vascular tone, but persistent or excessive adrenergic signaling may contribute to arrhythmia, metabolic stress, receptor desensitization, and microcirculatory dysfunction.^15^

The baroreflex is a key mechanism for short-term hemodynamic stability. Baroreceptors in the carotid sinus and aortic arch detect arterial stretch and transmit afferent signals to brainstem cardiovascular centers, which adjust heart rate, cardiac function, and vascular resistance through sympathetic and parasympathetic efferents.^16^ Concurrent HPA-axis activation potentiates these sympathetic effects via glucocorticoid-mediated modulation of vascular reactivity and metabolic substrate availability, creating an integrated stress response [Figure 1].

**Figure 1:**
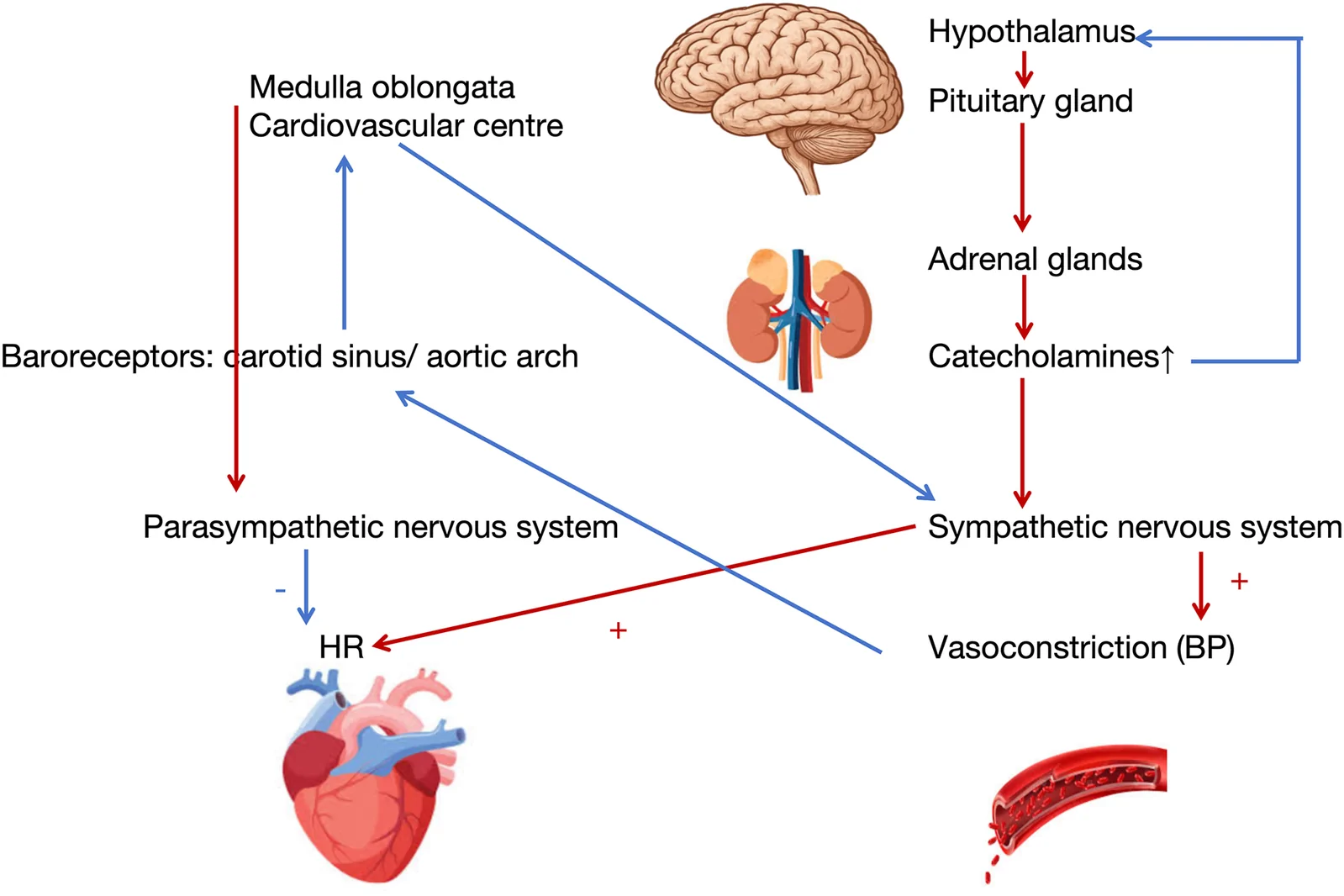
Autonomic and neuroendocrine regulation of cardiovascular homeostasis. This original conceptual illustration depicts baroreceptor input from the carotid sinus and aortic arch to medullary cardiovascular centers, with sympathetic and parasympathetic outputs affecting heart rate, myocardial contractility, and vascular tone (thereby influencing blood pressure). It also shows hypothalamic-pituitary-adrenal signaling through the pituitary and adrenal glands, catecholamine release, and a feedback relationship with hypothalamic control. The plus and minus symbols indicate the positive or negative influences shown in the schematic. BP: Blood pressure; HR: Heart rate.

## Autonomic nervous system (ANS) dysfunction and the host-organ unregulated response (HOUR) framework

Critical illness can follow infection, trauma, pancreatitis, major surgery, cancer-related complications, or other severe insults. The host-organ unregulated response (HOUR) framework integrates autonomic, neuroendocrine, inflammatory, immune, coagulation, metabolic, bioenergetic, and microcirculatory disturbances that may connect an initiating insult to hemodynamic instability and organ dysfunction.^17^^,^^18^ It provides a conceptual approach for considering the interactions of these processes during the evolution of critical illness.

In this review, HOUR is used as an integrative framework to organize the interacting pathophysiological processes discussed below.

Observational intensive care unit (ICU) studies describe hyperdynamic cardiac phenotypes during sepsis, and physiological work links excessive cardiac and respiratory drive with secondary organ stress.^19^^,^^20^ These findings support sympathetic activation as one contributor to, rather than the sole cause of, secondary organ stress. Despite these findings, adrenergic excess remains a significant contributor to tachycardia, arrhythmia, increased myocardial oxygen demand, vascular dysregulation, and impaired microcirculatory function. Increased pulmonary blood flow and transpulmonary pressure may aggravate pulmonary endothelial injury and edema,^21^ whereas endothelial and mitochondrial injury may amplify organ dysfunction.^22^ Prolonged catecholamine exposure can also promote β-adrenergic receptor desensitization and reduced responsiveness.^23^

In critical illness, reduced parasympathetic activity may decrease vagal acetylcholine signaling, thereby removing inhibition of nuclear factor kappa-B (NF-κB) activation in macrophages and facilitating pro-inflammatory mediator release.^24^ Experimental studies of the cholinergic anti-inflammatory pathway further support interactions among vagal signaling, inflammation, coagulation, and organ injury.^25^^,^^26^ These mechanisms are biologically plausible contributors to a cycle of autonomic imbalance and systemic inflammation, but their therapeutic implications in critically ill cancer patients remain to be determined.

## Systemic and organ manifestations of autonomic nervous system dysfunction in critically ill cancer patients

In critically ill cancer patients, ANS dysfunction is clinically important because malignancy and anticancer therapy can turn physiological stress responses into persistent dysautonomia. The resulting syndrome may include impaired blood-pressure buffering, vasopressor hyporesponsiveness, microcirculatory flow heterogeneity, metabolic instability, and organ-specific perfusion failure. Organ-level consequences are summarized in Table 1, and cancer-specific differences from general critical illness are summarized in Table 2.

**Table 1:** Representative organ manifestations and monitoring variables associated with autonomic dysfunction.

Target organ	Representative pathophysiological change	Potential clinical or monitoring variable	Evidence source	Representative references
Heart	Tachycardia, arrhythmia, increased myocardial oxygen demand, and β-adrenergic receptor desensitization	HRV recorded over a clearly reported duration; rhythm; cardiac function; perfusion variables	Observational ICU studies and mechanistic reviews	19,20,23,27
Lung	Increased pulmonary blood flow or transpulmonary pressure with endothelial injury and edema	Pulmonary blood flow; transpulmonary pressure; extravascular lung water	Clinical hemodynamic study	21
Brain	Impaired cerebrovascular autoregulation	PRx and patient-specific MAP range; interpret according to monitoring context	Neurocritical-care cohorts and methodological studies	28,29
Kidney	Reduced renal perfusion and venous-congestion effects on renal blood flow	Renal perfusion pressure and tissue perfusion trends; no universal ANS-specific cutoff	Physiological and perioperative/ICU observational studies	30,31
Gastrointestinal tract	Potential gastrointestinal dysmotility and perfusion impairment	Clinical perfusion assessment; intra-abdominal pressure where indicated; no validated ANS-specific cutoff	Indirect neurocritical-care and physiological evidence	32

The references presented in this table were selected as representative examples of the organ-specific mechanisms and monitoring variables discussed in the main text. Selection considered direct relevance to the target organ or parameter, study design, population applicability, and recency. Because the table incorporates heterogeneous clinical, observational, methodological, and physiological evidence, no formal evidence-grading system was applied. The references are illustrative rather than exhaustive. ANS: Autonomic nervous system; HRV: Heart rate variability; ICU: Intensive care unit; MAP: Mean arterial pressure; PRx: Pressure reactivity index.

**Table 2:** Contextual comparison of autonomic dysfunction in general critical illness and critically ill cancer patients.

Characteristic	General critical illness	Critically ill cancer patients
Contributing mechanisms	Acute inflammation, autonomic stress responses, and treatment-related physiological disturbances	Acute critical illness superimposed on malignancy, anticancer treatment, cachexia, endocrine disturbance, or immune-mediated toxicity
Potential etiologies	Sepsis, trauma, surgery, cardiac arrest, and other acute insults	Direct tumor effects; paraneoplastic disease; chemotherapy, radiotherapy, ICI toxicity^34^^,^^35^; CAR-T toxicity^36^; or BiTE toxicity^33^; metabolic and endocrine abnormalities
ANS phenotype	Sympathetic activation and/or parasympathetic withdrawal; phenotype varies with cause and phase	Mixed or global autonomic impairment may occur; direct ICU prevalence data are limited
Cardiovascular manifestations	Tachycardia, arrhythmia, blood-pressure instability, or vasopressor dependence	Similar manifestations with additional cancer- and treatment-specific differential diagnoses
Gastrointestinal manifestations	Ileus, gastroparesis, or stress-related dysmotility	Dysmotility may also reflect paraneoplastic, immune-mediated, drug-related, infiltrative, or metabolic causes
Other manifestations	Thermoregulatory, sudomotor, pupillary, urinary, or neurological abnormalities may occur	The same manifestations require assessment for malignancy- and treatment-specific causes
Reversibility	Depends on etiology, illness severity, and treatment response	May be reversible, persistent, or progressive; cancer-specific prospective data are insufficient
Prognostic evidence	Reduced autonomic variability is associated with adverse outcomes in several ICU cohorts	Independent prognostic value in acutely critically ill cancer patients has not been established

This table presents a conceptual comparison and is not intended as an exhaustive evidence synthesis. ANS: Autonomic nervous system; BiTE: Bispecific T-cell engager; CAR-T: Chimeric antigen receptor T-cell; ICI: Immune checkpoint inhibitor.

Available studies suggest that autonomic impairment is common in patients with advanced malignancy and in cancer survivors exposed to cardiotoxic or neurotoxic therapies.^37^^,^^38^ These findings support a high clinical suspicion for ANS dysfunction in patients presenting with refractory hypotension, unexplained tachyarrhythmia, gastrointestinal dysmotility, thermoregulatory disturbance, or prolonged dependence on vasopressors in oncocritical care.

Cancer-associated autonomic injury may arise from paraneoplastic, treatment-related, immune-mediated, or direct neurological mechanisms. Autoimmune autonomic ganglionopathy with ganglionic α3-nicotinic acetylcholine-receptor antibodies is occasionally paraneoplastic and has been reported with small-cell carcinoma and thymoma.^39^ Central nervous system metastases or leptomeningeal disease may also disrupt autonomic pathways when relevant structures are involved. Separately, lung adenocarcinoma has been shown experimentally to activate vagal sensory neurons and increase sympathetic efferent signaling in the tumor microenvironment.^40^

Anticancer therapies may affect autonomic pathways through several mechanisms. Anthracycline-associated reductions in HRV have been reported in prediction and survivorship studies, but these findings demonstrate association rather than acute causality in critically ill patients.^41^^,^^42^ Taxane- and carboplatin-associated changes in HRV have also been described,^43^ and bortezomib has been associated with treatment-emergent autonomic neuropathy.^44^ Evidence for direct autonomic toxicity from newer targeted therapies in the ICU remains limited. Related cardiovascular or neurological toxicities should be interpreted alongside evidence of autonomic involvement.

Radiotherapy-related autonomic dysfunction has been described mainly in long-term follow-up studies and case series. Head and neck irradiation may injure the carotid sinus, baroreceptor afferents, or vagal pathways, leading to orthostatic hypotension, blood-pressure lability, paroxysmal hypertension, inappropriate tachycardia, and impaired baroreflex sensitivity (BRS).^45^ Chest irradiation may be associated with reduced cardiac vagal tone and decreased HRV, whereas abdominal or pelvic irradiation may affect sympathetic chains or enteric plexuses and contribute to gastrointestinal dysmotility and impaired visceral autonomic regulation. However, direct evidence in acutely critically ill cancer patients is scarce. Whether radiotherapy-related autonomic injury contributes to hemodynamic instability in the ICU therefore remains an important knowledge gap in oncocritical care.

Microcirculatory and metabolic dysfunction may amplify cancer-associated dysautonomia. Malignancy-related inflammation, endothelial injury, hypercoagulability, cachexia, exogenous corticosteroid exposure, and treatment-related vascular toxicity may contribute to microcirculatory dysfunction, including heterogeneous capillary perfusion, impaired oxygen extraction, capillary leak, and microthrombosis.^46^^,^^47^ Autonomic pathways participate in thermoregulatory control.^48^ Acute stress may also contribute to stress hyperglycemia in critically ill patients.^49^

Immune checkpoint inhibitor (ICI)-related autonomic neuropathy is thought to result from aberrant T-cell activation and immune-mediated injury to autonomic ganglia or peripheral autonomic nerves. Clinical manifestations may include orthostatic hypotension, gastrointestinal dysmotility, arrhythmia, thermoregulatory disturbance, and hemodynamic collapse.^34^^,^^35^ These presentations may mimic sepsis-associated dysautonomia, but management differs because immune-mediated toxicity may require immunomodulatory therapy rather than infection control alone.

Management of suspected ICI-related or paraneoplastic autonomic neuropathy requires prompt recognition, interruption of the offending therapy when appropriate, hemodynamic support, and early multidisciplinary discussion. Glucocorticoids are commonly used for immune-mediated cases, and refractory presentations may require intravenous immunoglobulin, plasma exchange, or other immunosuppressive strategies.^35^^,^^39^

## Evaluation methods for autonomic nervous system function

Assessment of the autonomic nervous system in critical illness focuses on cardiovascular autonomic regulation and blood-flow autoregulation. Direct neural recordings and serial catecholamine measurements are generally impractical for routine ICU use. Available approaches include HRV, BPV, BRS, and standardized cardiovascular autonomic reflex tests; feasibility and interpretation depend on rhythm, ventilation, medications, consciousness, and hemodynamic stability.

The Valsalva maneuver produces four characteristic hemodynamic phases (phase II comprises early and late components) during standardized forced expiration, commonly at 40 mmHg for 15 s.^50^ Phase I is a transient mechanical increase in blood pressure; early phase II reflects reduced venous return and stroke volume; late phase II reflects sympathetic vasoconstriction and pressure recovery; phase III is a transient pressure fall after release; and phase IV normally includes a pressure overshoot with reflex bradycardia. Interpretation requires continuous beat-to-beat pressure and electrocardiographic recording, and results are affected by age, position, medications, effort, and cardiac function. The key Valsalva maneuver assessment parameters are shown in Table 3.

**Table 3:** Key Valsalva maneuver assessment parameters.

Parameter	Definition	Interpretation in a standardized test
Early phase II	Maximum blood-pressure decline during strain	The magnitude is age- and protocol-dependent; a fixed universal cutoff should not be applied in isolation
Late phase II	Blood-pressure recovery during continued strain	Recovery toward baseline is expected; reduced or absent recovery supports adrenergic impairment
Phase IV overshoot	Peak systolic blood pressure after release	An overshoot above baseline is expected in a typical response; absence may indicate adrenergic impairment
Systolic pressure recovery time	Interval from the phase III nadir until systolic pressure returns to baseline	<4 s is commonly used as a laboratory reference; interpretation remains protocol-dependent
Valsalva ratio (RR interval)	Longest RR interval after release divided by the shortest RR interval during strain	Interpret using age-adjusted laboratory norms; 1.21 is a commonly cited lower reference, not a universal cutoff
Valsalva ratio (heart rate)	Ratio of peak HR to trough HR	≥1.21

Values and waveform morphology should be interpreted using a standardized protocol and age-adjusted laboratory reference data.^50^ Pulse-pressure decline should be interpreted in relation to the testing protocol, age, and baseline hemodynamics; a universal cutoff should not be applied. BP: Blood pressure; HR: Heart rate; RR: Interval between consecutive R waves.

Orthostatic testing evaluates cardiovascular responses to upright posture. For conventional bedside assessment, blood pressure and heart rate are recorded after at least 5 min supine and after standing; head-up tilt is used when active standing is not feasible or when more detailed evaluation is required. Test angle, duration, continuous monitoring, and symptom correlation should be reported.

Classic orthostatic hypotension is defined as a sustained reduction in systolic blood pressure of at least 20 mmHg or diastolic blood pressure of at least 10 mmHg within 3 min of standing or head-up tilt to at least 60°.^51^ In patients with marked supine hypertension, larger thresholds may be appropriate. Results must be interpreted in clinical context because hypovolemia, medications, deconditioning, arrhythmia, and cardiac dysfunction can mimic or modify neurogenic responses.

### Cardiovascular variability metrics

HRV quantifies the variation in intervals between consecutive normal sinus beats. HRV is influenced by both branches of the ANS, respiratory pattern, circadian effects, age, medications, ectopy, pacing, and illness severity.^27^ Reduced HRV has prognostic associations in several cardiovascular and critical-care populations,^52^^,^^53^ but these associations do not establish a single diagnostic threshold for dysautonomia in critically ill cancer patients.

#### Heart-rate variability: time-domain analysis

Time-domain analysis includes the standard deviation of all normal-to-normal intervals (SDNN) and related indices. Recording duration must be reported: values derived from 24-h Holter monitoring are not interchangeable with those from short resting recordings. Historical SDNN cutoffs of <50 ms and >100 ms were derived largely from 24-h recordings in post-myocardial-infarction populations^54^ and should not be treated as validated diagnostic thresholds in critically ill cancer patients.

#### Heart-rate variability: frequency-domain analysis

Frequency-domain analysis partitions RR-interval variability into frequency bands. High-frequency power is strongly influenced by respiration and vagal modulation, whereas low-frequency power reflects combined autonomic and baroreflex influences.^55^ The low-frequency-to-high-frequency (LF/HF) ratio has historically been used as a surrogate of sympathovagal balance, but it should not be interpreted as a direct or universal measure of sympathetic-to-parasympathetic activity, particularly in mechanically ventilated or critically ill patients.^56^

#### Blood-pressure variability

BPV describes the magnitude and temporal pattern of blood-pressure fluctuations over beat-to-beat, short-term, circadian, or visit-to-visit intervals. These time scales have different determinants, including autonomic activity, respiration, vascular properties, behavior, medications, and measurement technique.^57^

Frequency-domain BPV can characterize rhythmic pressure oscillations, but individual bands do not map exclusively to one autonomic branch. HRV, BPV, and BRS should therefore be interpreted jointly as cardiovascular variability metrics, with explicit reporting of acquisition duration, sampling rate, preprocessing, ventilation, rhythm, and vasoactive medication exposure.

### Baroreflex sensitivity (BRS)

BRS quantifies the relationship between spontaneous or induced changes in blood pressure and the subsequent RR-interval response. Sequence and spectral methods report BRS in ms/mmHg, which depend on the method, age, rhythm, respiration, and clinical state. Fixed cutoffs should therefore not be used without population- and method-specific validation.^58^

Sequence and spectral analysis techniques are used to assess BRS. Frequency-domain analysis of BRS considers oscillations in the low-frequency band of 0.04–0.15 Hz and high-frequency band of 0.15–0.40 Hz. Transfer function analysis quantifies how different frequencies and amplitudes of blood pressure are transmitted to the RR interval, including coherence, phase, and gain. Coherence measures the strength of the relationship between blood pressure and RR interval oscillations and is analogous to the correlation coefficient *r* in linear regression. *Phase* shift (ϕ, −π to π) quantifies the temporal lag between blood pressure and RR interval oscillations, with negative values indicating baroreflex-mediated RR changes following blood-pressure variations. *Gain* (ms/mmHg), calculated as $\sqrt{{RR}_{power}/{BP}_{power}}$ in coherent (≥0.5) and negatively phased frequency bands, quantifies the BRS by relating changes in the RR interval to variations in blood pressure.

## High-resolution multimodal monitoring of blood-flow autoregulation

Blood-flow autoregulation allows organs to maintain stable perfusion across a range of arterial pressures. During shock, vital organs such as the heart, brain, and kidneys maintain adequate blood supply through robust autoregulatory mechanisms, whereas skeletal muscle and the gastrointestinal tract, which have weaker autoregulatory capacity, are more vulnerable to ischemic injury.

High-resolution monitoring captures continuous physiological waveforms and synchronizes signals from multiple sensors, allowing detection of transient hemodynamic changes and dynamic assessment of organ perfusion. Its value lies in the temporal alignment and integrated interpretation of signals rather than in a single mandatory sampling frequency.

High-resolution multimodal monitoring (MMM) refers to the synchronized acquisition and integration of physiological signals at sampling frequencies appropriate to the waveform being analyzed. Components may include continuous arterial pressure, electrocardiography (ECG), cerebral oximetry, transcranial Doppler, intracranial pressure (ICP) where indicated, and tissue-perfusion measures. Raw ECG and pressure-waveform analysis generally require substantially higher sampling rates than intermittently recorded clinical variables. Data quality, artifact handling, time synchronization, storage, and prespecified analytical methods should be reported.

Organ perfusion pressure (OPP), defined as arterial pressure minus venous pressure (Pa − Pv), is a fundamental determinant of organ blood flow and is influenced by arterial pressure, venous or tissue pressure, and vascular resistance. Low perfusion pressure can lead to organ ischemia, whereas excessively high pressure may cause overperfusion injury. Critical closing pressure (Pcrit), the minimum pressure required to maintain vascular patency, is directly related to vascular resistance: elevated sympathetic tone and catecholamine release increase Pcrit through excessive vasoconstriction, raising the arterial pressure threshold needed to sustain perfusion and predisposing to ischemic organ damage.

MMM enables analysis of beat-to-beat arterial pressure waveforms and real-time calculation of cardiac output (CO), mean arterial pressure (MAP), systemic tissue perfusion pressure (TPP = MAP − Pcrit), and Pcrit by extrapolating the CO–MAP relationship to zero flow. Simultaneous Doppler assessment of organ-specific blood flow (e.g., middle cerebral, renal, and brachial arteries), when integrated with invasive arterial pressure monitoring, allows determination of organ-level Pcrit as the intercept point at which arterial flow ceases during MAP reduction.^30^^,^^31^ During sympathetic hyperactivity, systemic vasoconstriction elevates Pcrit, compromising tissue perfusion and necessitating prompt intervention with optimized sedation, analgesia, and stress reduction strategies to prevent secondary organ injury.

Brain multimodal monitoring integrates real-time measurements of cerebral blood flow (CBF), intracranial pressure (ICP), brain tissue oxygen partial pressure (PbtO2), and electroencephalography (EEG) with systemic hemodynamics (arterial pressure and heart rate) to detect early pathophysiological disturbances and mitigate secondary brain injury. Cerebral autoregulation can be quantitatively assessed through continuous beat-to-beat arterial pressure and intracranial pressure monitoring, in which the pressure reactivity index (PRx, the moving Pearson correlation coefficient between slow-wave ICP and MAP over 200–300 s windows) indicates intact autoregulation when PRx is < 0.2. Transfer function analysis of these signals further characterizes autoregulatory dynamics through phase differences and gain, with reduced phase shifts signifying impaired regulation. The lower and upper limits of the autoregulatory curve demarcate the functional MAP range in which CBF remains autoregulated and relatively constant despite changes in perfusion pressure; MAP-dependent CBF changes outside this range reflect autoregulatory failure. Moving-window correlation analysis across MAP ranges identifies the optimal blood pressure (lowest PRx) for maintaining autoregulation and guiding individualized hemodynamic management in neurocritical care.^28^

Maintaining MAP above patient-specific optimal levels enhances organ perfusion and oxygen delivery while helping prevent dysfunction. Emerging technologies such as Doppler ultrasound and near-infrared spectroscopy (NIRS) now allow real-time assessment of autoregulation in multiple organ systems (renal and peripheral) through continuous monitoring of blood flow and tissue oxygenation, supporting precise blood pressure titration and targeted management of sympathetic hyperactivity to mitigate secondary organ injury.^29^

Most evidence supporting high-resolution MMM and AI-assisted physiological assessment comes from general ICU or neurocritical-care populations. Cancer-specific evaluation is needed because malignancy, cachexia, cardiotoxic therapies, immune-related adverse events, metabolic disturbances, rhythm abnormalities, and medications may alter physiological signals. Alternative autoregulation indices, including the cerebral oximetry index and transcranial Doppler-derived Mx, warrant evaluation in oncocritical care.

## Clinical management and therapeutic strategies for cancer-associated dysautonomia in the intensive care unit (ICU)

Clinical management should begin with confirmation of the autonomic phenotype, exclusion of reversible mimics, treatment of the underlying cause, and individualized organ support. Analgesia and sedation may be appropriate when pain, agitation, or excessive respiratory or cardiac drive are present. Monitored sympatholysis and disease-specific immunomodulation should be considered in selected patients after evaluation of contraindications and diagnostic certainty. The potential management domains are shown in Figure 2, and the stepwise clinical algorithm is shown in Figure 3.

**Figure 2:**
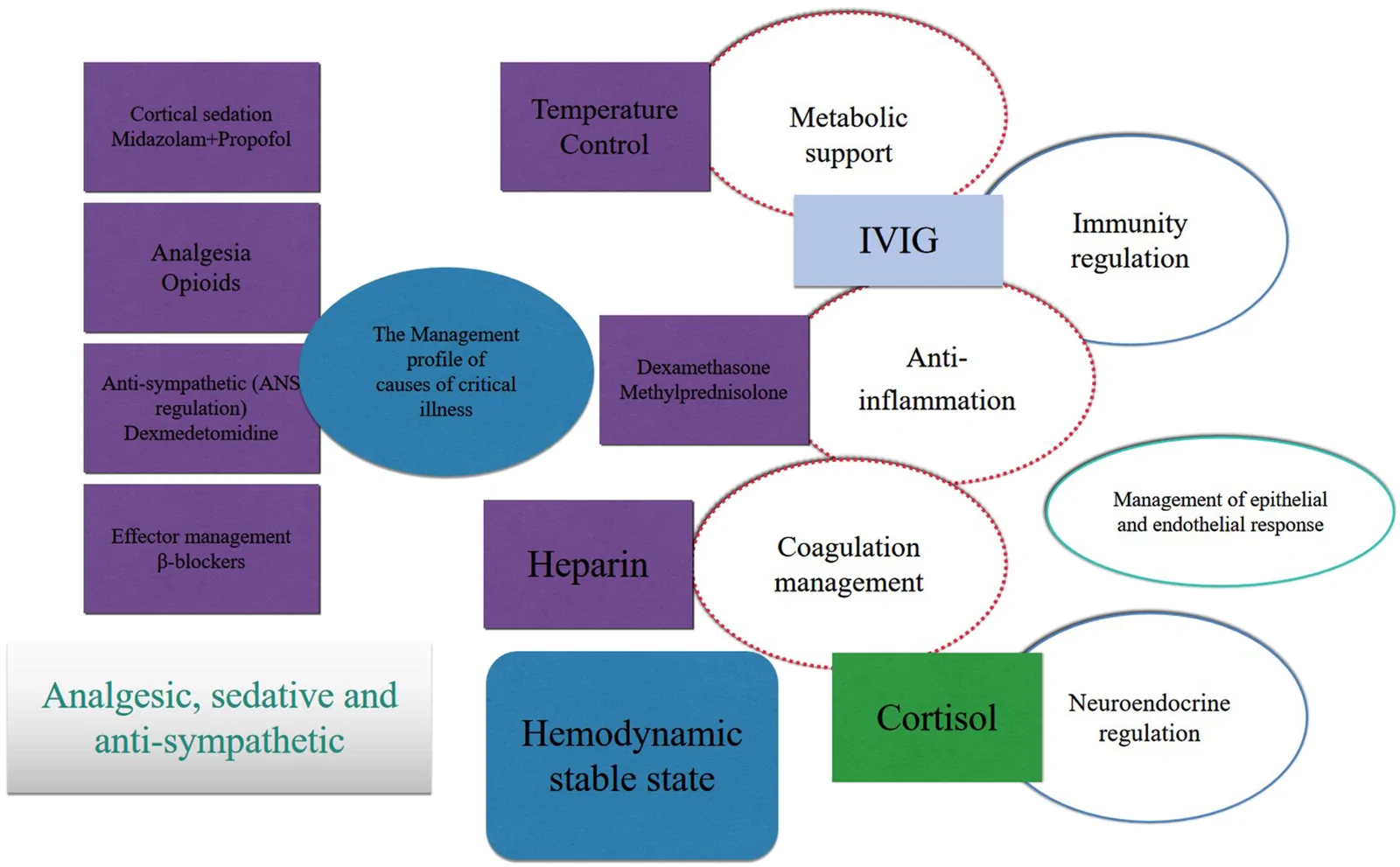
Potential management domains for autonomic nervous system dysfunction and the host-organ unregulated response in critically ill patients. This conceptual illustration groups potential management domains around the cause of critical illness and hemodynamic stability. The left side highlights cortical sedation, opioid analgesia, anti-sympathetic therapy with dexmedetomidine, and β-blockers. The central and right-sided domains include temperature control, metabolic support, IVIG, anti-inflammation with dexamethasone or methylprednisolone, coagulation management with heparin, immunity regulation, neuroendocrine regulation with cortisol, and epithelial or endothelial responses. These domains are potential intervention or monitoring considerations; their selection and intensity should be individualized according to the underlying cause, hemodynamics, organ function, and adverse-effect risk. This is an original conceptual illustration created by the authors based on the literature cited in the main text. IVIG: Intravenous immunoglobulin.

**Figure 3:**
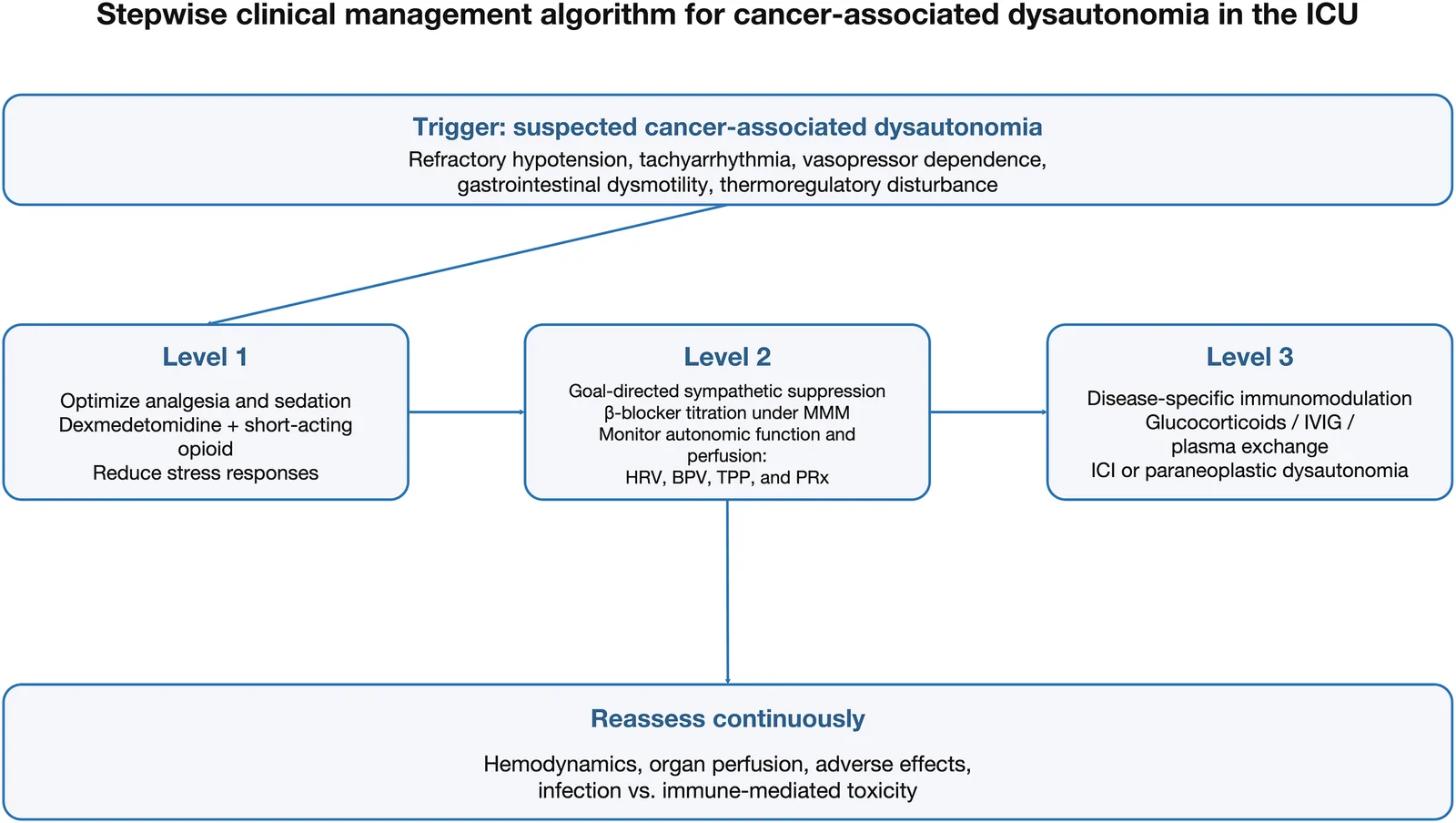
Stepwise clinical management algorithm for cancer-associated dysautonomia in the intensive care unit (ICU). This original conceptual illustration presents a stepwise algorithm for suspected cancer-associated dysautonomia. The trigger box lists refractory hypotension, tachyarrhythmia, vasopressor dependence, gastrointestinal dysmotility, and thermoregulatory disturbance. Level 1 emphasizes optimizing analgesia and sedation, including dexmedetomidine plus a short-acting opioid, and reducing stress responses (e.g., ReMiProDE protocol). Level 2 proposes the titration of β-blockers under multimodal monitoring (MMM) and ANS monitoring (e.g., heart rate variability (HRV), blood pressure variability (BPV), tissue perfusion pressure [TPP] or pressure reactivity index [PRx]). Level 3 reserves disease-specific immunomodulation (glucocorticoids, intravenous immunoglobulin [IVIG], or plasma exchange) for selected immune checkpoint inhibitor (ICI)-related or paraneoplastic cases. Hemodynamics, organ perfusion, adverse effects, and infection vs immune-mediated toxicity should be reassessed continuously. ANS: Autonomic nervous system; ReMiProDE: Remifentanil, midazolam, propofol, dexmedetomidine, and esmolol.

The authors’ group has proposed a goal-directed sedation-analgesia-anti-sympathetic strategy termed remifentanil, midazolam, propofol, dexmedetomidine, and esmolol (ReMiProDE) for patients with marked stress and sympathetic activation.^59^ ReMiProDE incorporates remifentanil, midazolam, propofol, dexmedetomidine, and individualized β-blockade (esmolol). When deep sedation is clinically indicated, a bispectral index or entropy target of 40–60 may be used to guide sedation depth.

ReMiProDE provides a goal-directed approach to analgesia, sedation, and sympathetic modulation in patients with marked stress activation. Drug selection, dose, depth of sedation, and use of β-blockade should be individualized according to the clinical indication, hemodynamics, organ function, concomitant medications, and adverse-effect risk.

The management goals are to control pain and agitation, attenuate excessive stress responses, preserve adequate perfusion, and continuously reassess the diagnosis and treatment response.

## Application of artificial intelligence in the evaluation of autonomic nervous system function

Recent advances in machine learning have significantly improved our ability to analyze physiological signals, particularly HRV, BPV, and the cerebral perfusion pressure index. These signals serve as critical biomarkers for the function of the ANS and cerebrovascular autoregulation. Traditional methods often struggle with the high dimensionality, nonlinearity, and noise inherent in these signals, whereas machine learning algorithms excel in feature extraction and predictive modeling. Key barriers to clinical implementation include data standardization and interoperability among monitoring platforms.

Machine-learning methods can integrate nonlinear and time-dependent physiological features, but performance depends on cohort definition, signal quality, preprocessing, calibration, and external validation. Interpretation of HRV, BPV, or PRx requires models developed and validated for the corresponding physiological signals and target population.

Current applications include risk prediction and physiological time-series analysis, although use in relevant ICU populations requires further evaluation. In a retrospective Medical Information Mart for Intensive Care IV (MIMIC-IV) cohort, ChronoSynthNet used routine electronic-health-record time-series data from 1968 patients with septic shock to predict hypotension and norepinephrine infusion rate; it achieved an internal-test area under the receiver operating characteristic curve (AUROC) of 0.89 (95% confidence interval [CI], 0.836–0.938), specificity of 97%, precision of 92%, and recall of 74%, with a median hypotension-prediction lead time of 3.5 h.^60^ Its transportability to cancer-associated dysautonomia and prospective multicenter ICU use warrants further evaluation. Separately, 29 cardiovascular autonomic indices were extracted from resting ECG and continuous blood-pressure recordings in 1060 healthy participants; selected features were then entered into a Gaussian-process regression model. In a selected comparison, the high-risk group had a mean cardiovascular autonomic age gap of +11.1 years versus −0.9 years in the low-risk group, with cardiovascular autonomic age correlating with Framingham risk (*r* = 0.42; *P* < 0.001).^61^ In healthy volunteers, these findings provide a physiological reference; their applicability to ICU and cancer populations requires further evaluation. Further development should include external and prospective validation, robust waveform preprocessing and signal-quality assessment, model calibration, clinical-utility evaluation, and regulatory assessment.^62^

Several technical limitations apply to AI-based autonomic assessment in critically ill cancer patients. Cachexia, cardiotoxic therapy, pacing, arrhythmia, mechanical ventilation, vasoactive medication, sedation, and metabolic disturbance may confound conventional variability indices. Heterogeneity in tumor type, stage, treatment cycle, immune status, and comorbidity further limits generalizability. Future cancer-specific models should combine prespecified oncological features with high-resolution physiological data and undergo external, prospective, and clinical-utility validation before deployment.

## Future directions and unmet needs

The intersection of autonomic neuroscience, oncology, and critical care presents several priorities. Prospective studies should establish incidence and trajectories of autonomic dysfunction by cancer type, disease stage, treatment, and critical-illness phenotype. Reproducible ICU-compatible autonomic assessment protocols are needed, with explicit reporting of signal acquisition, ventilation, rhythm, sedation, and vasoactive therapy. Interventional studies should test whether autonomic-guided management improves patient-centered outcomes. Cancer-specific multimodal AI models require prospective evaluation in real-world ICU settings, and long-term autonomic outcomes after critical illness remain underexplored.

## Study limitations

This review has several limitations. Direct evidence in acutely critically ill cancer patients remains limited, particularly for radiotherapy-related injury, targeted therapies, high-resolution MMM, AI-assisted prediction, and autonomic-guided intervention. Many mechanistic links are extrapolated from general ICU, neurocritical-care, perioperative oncology, cancer-survivorship, or physiological populations and would benefit from cancer-specific prospective studies.

## Summary

This review summarizes ANS dysfunction in critically ill cancer patients and its potential hemodynamic and organ-perfusion implications. HOUR is used as an integrative framework for the interacting autonomic, neuroendocrine, inflammatory, immune, coagulation, metabolic, bioenergetic, and microcirculatory disturbances discussed in this review. Conventional cardiovascular autonomic tests and emerging MMM or AI methods may provide complementary information, and their interpretation should account for measurement context and population-specific factors. Management should prioritize the underlying cause, individualized supportive care, and disease-specific therapy. Autonomic-targeted interventions should be evaluated prospectively in critically ill cancer patients.

## Authors contribution

Xiaoting Wang: Conceptualization, methodology, supervision, funding acquisition, and final review and approval. Shiyi Gong: Original draft writing and visualization. Yun Chu: Original draft writing and editing. All the authors critically revised and approved the final version of the manuscript.

## Ethics statement

Not applicable.

## Data availability statement

No new datasets were generated or analyzed in this narrative review. All cited evidence is available from the published literature.

## Declaration of generative AI and AI-assisted technologies in the writing process

During the preparation of this work, the authors used a large language model to assist with English language editing. This model was used under the direct supervision of the corresponding author. The authors take full responsibility for the content of the publication, including all analyses, interpretations, and conclusions.

## Funding

This work was supported by the Noncommunicable Chronic Diseases-National Science and Technology Major Project (Grant No. 2024ZD0526100) and CAMS Innovation Fund for Medical Science (CIFMS) (Grant No. 2024-I2M-C&T-B-003).

## Conflict of interest

The authors declare that they have no known competing financial interests or personal relationships that could have appeared to influence the work reported in this paper.

## Appendix A Supplementary data

Supplementary Table 1 summarizes representative clinical studies included in this narrative review.
