# Supplementary material for: Autonomic nervous system dysfunction in critically ill cancer patients: Hemodynamic consequences and multimodal assessment strategies
Source: Cancer Pathog Ther. 2026 Oct 21;4(6):471–9. doi: 10.1016/j.cpt.2026.08.001 (PMC13717020; doi:10.1016/j.cpt.2026.08.001)
Supplement: Supplementary file 1 [file mmc1.docx]

**Supplementary Table 1**

Representative clinical studies included in this narrative review

| Study year | First author | Study design | Sample size | Patient population | ANS assessment method | Prevalence of ANS dysfunction | Key prognostic finding | Representative reference |
| --- | --- | --- | --- | --- | --- | --- | --- | --- |
| 2024 | Amit Patel | Systematic review and meta-analysis | 9 included studies | Postoperative and ICU patients | HRV (SDNN, RMSSD, LF, HF, LF/HF ratio) | Not directly reported | Reduced SDNN is significantly associated with delirium; other HRV indices show weak or no association | ^1^ |
| 2023 | Satomoto M | Prospective cohort | 36 (14 delirium/22 non-delirium) | Patients ≥ 65 years undergoing elective cardiac surgery | HRV (LF power, HF power, LF/HF ratio) | 38.9% (14/36) developed postoperative delirium | Preoperative lower HF power predicts postoperative delirium (*P* < 0.05) | ^2^ |
| 2023 | Li S | Retrospective cohort | 93 (33 Atrial fibrillation/60 Sinus Rhythm) | ICU patients | 20 HRV parameters (time-domain, frequency-domain, nonlinear) | Not reported | None of the HRV parameters was associated with 30-day all-cause mortality in patients with or without atrial fibrillation | ^3^ |
| 2021 | Echizen M | Prospective pilot study | 30 (7 delirium/23 non-delirium) | Patients undergoing esophageal cancer surgery | HRV (HF power) | 23.3% (7/30) developed postoperative delirium | Preoperative lower HF power was associated with postoperative delirium (*P* < 0.05) | ^4^ |
| 2023 | Hunakova L | Prospective cohort | 19 | COVID-19 patients | HRV (RMSSD, HF-HRV, 2UV%) | Not reported | Lower HRV indices correlate with higher inflammatory markers (CRP, IL-6), increased ICU admission risk, and longer hospital stay | ^5^ |
| 2023 | Lazar DR | Prospective cohort | 35 | Children with acute lymphoblastic leukemia | 24-hour Holter HRV (time-domain: SDNN, SDANN, rMSSD, pNN50; frequency-domain: LF, HF, LF/HF ratio) | HRV indices decreased after anthracycline exposure in this pilot cohort; effect estimates and measurement context should be interpreted from the source | HRV reduction precedes overt echocardiographic abnormalities; LF/HF ratio increase indicates early sympathetic dominance | ^6^ |
| 2023 | Liu J | Prospective cohort | 50 (19 docetaxel combined with carboplatin group/31 paclitaxel combined with carboplatin) | Patients with cervical cancer receiving taxane + carboplatin | HRV measurement | Not reported | Early detection of autonomic nerve damage by HRV | ^7^ |
| 2020 | Groehs RV | Prospective before-and-after observational study | 29 (12 5-FU/17 5-FU + oxaliplatin) | Patients with stage II-III colon adenocarcinoma after colectomy receiving adjuvant 5-FU or 5-FU + oxaliplatin | HRV, muscle sympathetic nerve activity, vascular endothelial function, and muscle blood flow | Not reported | Adjuvant 5-FU with or without oxaliplatin did not significantly change HRV, muscle sympathetic nerve activity, vascular endothelial function, or physical capacity | ^8^ |
| 2021 | Feng Y | Prospective cohort | 150 | *HER2*-positive breast cancer patients receiving trastuzumab | HRV (SDNN, RMSSD, DC) | 18.7% (28/150) developed trastuzumab-related cardiotoxicity | DC is an independent predictor of trastuzumab-related cardiotoxicity (AUC = 0.88), superior to baseline LVEF | ^9^ |
| 2020 | Ng DQ | Randomized controlled trial | 38 (21 cryotherapy/17 control) | Breast cancer patients receiving weekly paclitaxel | HRV, SSR, CIPN20 autonomic subscale | Cryotherapy was associated with differences in selected neuropathy outcomes; autonomic findings were exploratory | Cryotherapy was associated with lower CIPN20 autonomic scores and higher hand SSR amplitudes; these exploratory findings require confirmation | ^10^ |

The Table summarizes representative clinical studies and is not an exhaustive evidence table. Study design, population, measurement duration, effect estimates, and limitations should be interpreted from the cited source. All table citations are listed in the reference list below and are numbered independently from those in the main manuscript. The 2 UV%: Percentage of sequences of three cardiac periods with two unlike variations; ANS: Autonomic nervous system; AUC: Area under the curve; BPV: Blood pressure variability; BRS: Baroreflex sensitivity; CIPN20: European Organization for Research and Treatment of Cancer Quality of Life Questionnaire-Chemotherapy-Induced Peripheral Neuropathy 20; CRP: C-reactive protein; DC: Deceleration capacity; HF: High-frequency; HF-HRV: High-frequency heart rate variability; HRV: Heart rate variability; ICU: Intensive care unit; IL: Interleukin; LF: Low-frequency; LVEF: Left ventricular ejection fraction; pNN50: Percentage of adjacent normal-to-normal intervals differing by more than 50 ms; RMSSD: Root mean square of successive differences; SDANN: Standard deviation of the average normal-to-normal intervals over 5-minute segments; SDNN: Standard deviation of all normal-to-normal intervals; SSR: Sympathetic skin response.

References

1. Amit Patel K, Sethi A, Al Azazi E, McClurg C, Chowdhury T. The role of heart rate variability in predicting delirium: a systematic review and meta-analysis. *J Clin Neurosci.* 2024;124:122-129. https://doi.org/10.1016/j.jocn.2024.04.028

2. Satomoto M. Predicting postoperative emergence delirium from the heart rate variability of patients undergoing elective cardiac surgery. *Cureus.* 2023;15:e34613. https://doi.org/10.7759/cureus.34613

3. Li S, Yang Q, Wu P, et al. Heart rate variability parameters were not associated with 30-day all-cause mortality in intensive care unit patients with or without atrial fibrillation: a retrospective study of the MIMIC-IV database. *Shock.* 2023;60:24-33. https://doi.org/10.1097/SHK.0000000000002149

4. Echizen M, Satomoto M, Miyajima M, Adachi Y, Matsushima E. Preoperative heart rate variability analysis is as a potential simple and easy measure for predicting perioperative delirium in esophageal surgery. *Ann Med Surg (Lond).* 2021;70:102856. https://doi.org/10.1016/j.amsu.2021.102856

5. Hunakova L, Sabaka P, Zvarik M, et al. Linear and non-linear indices of vagal nerve in relation to sex and inflammation in patients with COVID-19. *Biomed Rep.* 2023;19:80. https://doi.org/10.3892/br.2023.1662

6. Lazar DR, Cainap S, Maniu D, et al. Anthracycline's effects on heart rate variability in children with acute lymphoblastic leukemia: early toxicity signs-pilot study. *J Clin Med.* 2023;12:7052. https://doi.org/10.3390/jcm12227052

7. Liu J, Guan W, Sun Y, et al. Early detection of the impact of combined taxane and carboplatin treatment on autonomic nerves in patients with cervical cancer: measurement of heart rate variability. *Front Physiol.* 2023;14:1126057. https://doi.org/10.3389/fphys.2023.1126057

8. Groehs RV, Negrao MV, Hajjar LA, et al. Adjuvant treatment with 5-fluorouracil and oxaliplatin does not influence cardiac function, neurovascular control, and physical capacity in patients with colon cancer. *Oncologist.* 2020;25:e1956-e1967. https://doi.org/10.1634/theoncologist.2020-0225

9. Feng Y, Qin Z, Yang Z. Deceleration capacity of heart rate predicts trastuzumab-related cardiotoxicity in patients with HER2-positive breast cancer: a prospective observational study. *J Clin Pharm Ther.* 2021;46:93-98. https://doi.org/10.1111/jcpt.13258

10. Ng DQ, Tan CJ, Soh BC, et al. Impact of cryotherapy on sensory, motor, and autonomic neuropathy in breast cancer patients receiving paclitaxel: a randomized controlled trial. *Front Neurol.* 2020;11:604688. https://doi.org/10.3389/fneur.2020.604688
